# Sirolimus-Eluting Electrospun-Produced Matrices as Coatings for Vascular Stents: Dependence of Drug Release on Matrix Structure and Composition of the External Environment

**DOI:** 10.3390/ma13122692

**Published:** 2020-06-12

**Authors:** Zhanna K. Nazarkina, Boris P. Chelobanov, Vera S. Chernonosova, Irina V. Romanova, Andrey A. Karpenko, Pavel P. Laktionov

**Affiliations:** 1 Institute of Chemical Biology and Fundamental Medicine, Siberian Branch, Russian Academy of Sciences, Novosibirsk 630090, Russia; boris.p.chelobanov@gmail.com (B.P.C.); vera_mal@niboch.nsc.ru (V.S.C.); irin-romanova@yandex.ru (I.V.R.); lakt@niboch.nsc.ru (P.P.L.); 2 Meshalkin National Medical Research Center, Ministry of Health of the Russian Federation, Novosibirsk 630055, Russia; andreikarpenko@rambler.ru

**Keywords:** sirolimus, electrospinning, drug release, polycaprolactone, 3D matrix, drug-eluting stents

## Abstract

Although a number of drug-eluting coatings for vascular stents (VSs) have been developed and are in commercial use, more efficient stent coatings and drug delivery systems are needed. Sirolimus (SRL) is a clinically important drug with antiproliferative and immunosuppressive activities that is widely used for coating stents. Here, we characterized SRL-enriched matrices, intended for coating vascular stents, that were produced by electrospinning (ES) on a drum collector from a solution of polycaprolactone (PCL) and human serum albumin (HSA), 1,1,1,3,3,3-hexafluoroisopropanol (HFIP), dimethyl sulfoxide (DMSO), and SRL. The release of tritium-labeled SRL (^3^H-SRL) from matrices in phosphate-buffered saline (PBS) or human blood plasma (BP) was studied. The introduction of DMSO in the ES blend decreased SRL release. The use of BP significantly accelerated SRL release through binding with serum biomolecules. The exchange of PBS or BP after every time point also increased SRL release. The maximum SRL release in BP was observed at 3 days. The matrices produced from the ES solution with DMSO and HSA released no more than 80% SRL after 27 days in BP, even under medium exchange conditions. Therefore, PCL-based matrices containing HSA, SRL, and DMSO can be used for coating VSs with prolonged SRL delivery.

## 1. Introduction

Stent implantation is one of the most common procedures for the reconstitution of blood flow through atherosclerosis-occluded arteries. The placement of the stent initiates a variety of reactions, including endothelial injury, crushing of the plaque, and stretching and lacerations of the arterial wall, which can lead to stent-induced restenosis [1]. These processes stimulate an inflammatory response—the predominant cause of neointimal proliferation and in-stent restenosis [2] accompanied by the migration and proliferation of smooth muscle cells (SMCs) and fibroblasts from the arterial wall [3]. It was suggested that neointima growth after application of angioplasty drug-eluting stents (DESs) could be prevented by reducing the growth and migration of SMCs and other cell types. Sirolimus (SRL)-eluting stents, which were approved in Europe in 2002 and in the United States in 2003 [4], are the most popular and have been shown to be the most effective among the relevant analogs in reducing coronary restenosis, re-intervention rates, and other adverse cardiac events in patients with coronary artery disease [5,6].

SRL (rapamycin) is a polyketide macrolide compound obtained by a strain of *Streptomyces hygroscopicus* [7]. In mammals, the molecular target of rapamycin, mTOR, is a cell cycle-specific kinase involved in complex intracellular signaling pathways that regulate cell growth and proliferation, immunity, angiogenesis, fibrogenesis, and metabolism [8]. After entering into the cell, SRL interacts with the immunophilin FKBP-12. This complex interacts with mTOR kinase and inhibits its activity [7]. Thus, SRL blocks the activation of p70S6 kinase, which leads to cell cycle arrest at the G1 to S phase.

SRL is a potent inhibitor of the antigen-induced T- and B-cells’ proliferation and antibody production [7], as well as the proliferation of a variety of cell types of nonlymphoid origin [9], including the inhibition of human coronary artery SMC proliferation [10]. Complete inhibition of PDGF-induced DNA synthesis in SMCs was achieved at 10 ng/mL SRL, and more than 50% inhibition was observed at an SRL concentration of 0.1 ng/mL. The inhibitory properties of SRL could be observed from a threshold concentration of 0.01 ng/mL. SRL is able to inhibit both quiescent and actively cycling cells [10].

SRL has a broad therapeutic window. Preclinical animal trials have shown that SRL demonstrates biological activity in doses from 18 to 1200 μg, without showing toxicity to the vessel wall [4]. Despite the promising pharmacological activities of SRL, however, its clinical application is complicated by poor aqueous solubility, first-pass metabolism, and nonspecific distribution in off-target sites [8]. SRL has some adverse, dose-dependent effects, including immunosuppression; the inhibition of bone growth; increased cholesterol, triglyceride, and creatinine serum levels; and decreased glomerular filtration rates. Leukopenia, thrombocytopenia, anemia, rash, stomatitis, arthralgia, diarrhea, hypertension, and hypokalemia may also occur [11]. Owing to its lipophilic nature, SRL is widely distributed in lipid membranes, resulting in a large volume of distribution and a relatively long half-life [12,13].

The most commonly used first-generation SRL-eluting stent in clinical practice is Cypher stent (SES; Cordis, Warren, NJ, USA) [14]. This DES consists of a stainless-steel platform with a strut thickness of 140 mm and 140 mg/cm^2^ sirolimus with a poly(ethylene-co-vinyl acetate) and poly(n-butyl methacrylate) polymer. A pharmacokinetic study showed that the maximum concentration of SRL in blood is observed between 3 and 4 h after stent implantation, with a peak concentration of 0.57 ng/mL in patients receiving one stent [15]. The average terminal-phase elimination half-life of SRL is 213 h.

It is known that SRL can induce fibrinoid vascular necrosis [16]. A reduction in the SRL dose can reduce this toxic effect in an animal model. A lower SRL dose (25, 40, and 100 µg), released from a nonpolymeric hydroxyapatite-coated stent built by MIV Therapeutics (Atlanta, GA, USA), results in less vascular healing, presenting significantly fewer fibrinoids without increasing neointima formation as compared with Cypher stent (111 µg of SRL) [16]. The REDOX trial showed that lower doses (40% or 70% compared with Cypher stent) of SRL effectively maintain luminal patency for up to at least 12 months, as determined at a follow-up [17].

The stent platform, the antiproliferative drug, and the drug carrier polymer are the three major components that determine stent safety and efficacy [18]. The Cypher stent has not been manufactured since 2011 because the first-generation stents displayed an increased risk for late thrombotic events [19]; this risk was considered to be associated with the permanent presence of the non-erodible polymers poly(ethylene-co-vinyl acetate) and poly(n-butyl methacrylate). Therefore, a second generation of DES was developed using more biocompatible polymer coatings. SRL was also used to develop a new generation of DESs with ultrathin (<70 μm) struts coated with biodegradable polymers, such as Orsiro and BioMime [20]. Another type of SRL-eluting stent is MiStent, which has ultrathin struts composed of crystalline SRL and a biodegradable polymer poly(lactic-co-glycolic acid). Clinical trials have shown the superiority of ultrathin strut biodegradable polymer-coated DESs compared with durable polymer-coated DESs in maintaining a good safety profile [21].

The Tetriflex (Sahajanand Medical Technology, Surat, India) is a new-generation, ultrathin (60 µm), biodegradable SRL-eluting stent with coating composed of a combination of hydrophilic and hydrophobic polymers for controlled and prolonged release of SRL. Clinical studies have demonstrated the safety and effectiveness of the Tetriflex stent [22].

One of the novel DESs is Svelte SRL-eluting stent containing fully bioresorbable polymer and pre-mounted on a single lumen, fixed-wire delivery system [23]. The pharmacokinetics of the Svelte stent mimics the SRL release of the Cypher stent. The Svelte amino-acid based polymer can be enzymatically degraded with complete resorption in 12 months. The OPTMIZE Trial, including 1630 subjects, will estimate the safety and clinical efficacy of the Svelte stent [23].

The DESs used in clinics are manufactured using different techniques for coating metallic vascular stents, including ultrasonic coating [24], dip-coating [25], spray-coating [2], air-brush [26], electrohydrodynamic jetting, plasma-treated coating, electrotreated coating [27], and electrospinning (ES) [28]. Coating techniques have an influence on drug release kinetics and clinical outcomes [27].

ES is based on the generation of polymer fibers from a blend of polymers and drugs in a strong electric field. The advantages of the ES method are the ability to obtain fibers of different diameters and ultrastructures laid in a 3D matrix, as well as its suitability for a broad range of materials, cost effectiveness, and mechanical support from the stented area that covers the inter-strut area (e.g., the InspireMD CGuard stents consisting of a fine mesh that inhibits neointima growth thanks to the prevention of cell migration from the damaged area) [29,30,31]. Drug release and dissolution of the bioactive substance can be controlled through the structure of polymer fibers, which is determined by the composition of the ES. In recent years, the ES technique and its modifications have been widely used in drug delivery and regenerative medicine. The FDA has approved heart valve prostheses and esophageal stent produced using the ES technique for clinical studies [32].

The materials for coating vascular stents (VSs) must be hemo- and biocompatible, and extensible by at least 2–2.5×. Synthetic materials produced from polycaprolactone (PCL) possess these properties and are widely used in medicine owing to their PCL biocompatibility, mechanical characteristics, and ease of manufacturing. The average degradation time for PCL is about 2–3 years [33]. The PCL matrices produced by ES are convenient for VSs owing to their mechanical properties [29] and good hemo- and biocompatibility [34,35]. The matrices produced from pure PCL possess poor wettability and low cell compatibility, but incorporation of human serum albumin (HSA) into the PCL matrices strongly interferes with its properties, thereby increasing the stiffness and biological properties of such matrices [29]. It is known that HSA reduces platelet adhesion and increases the thromboresistance and hemocompatibility of blood-exposed surfaces [36,37].

In a previous study of paclitaxel release from ES-produced matrices, we showed that drug release depends on the structure of the fibers and the composition of the surrounding medium [29].

In the current study, we investigated SRL release from ES-produced matrices using different solution compositions: 5% PCL/SRL/10% HSA, 5% PCL/SRL/10% HSA/3% dimethyl sulfoxide (DMSO), and 5% PCL/SRL. The physicochemical properties of the materials and the dependence of SRL release on the composition of the surrounding medium were also studied.

## 2. Materials and Methods

### 2.1. Production of ^3^H-Labeled Sirolimus

^3^H-SRL was synthesized by thermoactivated tritium exchange, as described earlier [38]. ^3^H-SRL was purified from byproducts using reversed-phase chromatography (RP-HPLC) on a C18 column using a gradient of acetonitrile in water (25–100%). The purity of ^3^H-SRL was evaluated by autoradiography following thin layer chromatography (TLC) on Kieselgel 60 F254 plates (Merck, Darmstadt, Germany, 25 Alufolien 20 cm × 20 cm) in a chloroform–methanol–water mixture (19:1:0.1, Rf = ~0.7). The radioactivity of the preparation was measured on a Tri-Carb 2800 TR β-counter (PerkinElmer, Waltham, MA, USA) in a “ULTIMA GOLD LTT” scintillator (Perkin Elmer, Waltham, MA, USA). The radioactivity of all samples was evaluated as reported earlier [29], that is, 0.1 mL of the sample was thoroughly mixed with 0.9 mL scintillator, and radioactivity was measured immediately.

### 2.2. Fabrication of the ES Matrices

The ES solutions were prepared using stock solutions of 9% PCL and 1% HSA (Sigma-Aldrich, St. Louis, MI, USA) in 1,1,1,3,3,3-hexafluoroisopropanol (HFIP, Sigma-Aldrich, USA). The HSA concentration in the matrices was 10% (w/w). SRL (150301, Fujian Kerui Pharmaceutical Co. Ltd., Fujian, China) was dissolved in HFIP or DMSO (Sigma-Aldrich, USA) and added to the ES solutions to a final concentration of 0.9 µg/cm^2^ (0.7 μg per disk). Then, 3% (v/v) DMSO was added to the solution of polymers. ^3^H-SRL was mixed with unlabeled SRL to reach radioactivity of 27,000 cpm/cm^2^ (21,000 per 10 mm diameter disk). To obtain more-uniform fibers, the PCL/SRL ES solution contained 0.1 mM triethylamine (TEA). To obtain the matrices containing ^3^H-SRL, we used a custom made ES device with an airproof chamber and an exhaust HEPA filter equipped with a Spellman SL 150 (30 kV, Spellman, Brockton, MA, USA) power supply. Matrices of 110–125 µm thickness were prepared using a drum collector (4 cm diameter and 5 cm length). Electrospinning conditions were the same for all matrices: voltage—26.5 kV; feed rate—1 mL/h; nozzle to collector distance—20 cm; collector rotation speed—300 rpm; 23–25 °C; 25–35% humidity. After fabrication, matrices were removed from the collector, dried in vacuum under 10 Pa for 12 h, and stored in sealed ziplock polyethylene containers at 4 °C.

### 2.3. Characterization of the Matrices

#### 2.3.1. Mechanical Characterization

Strain–stress diagrams were obtained using a universal Zwick/Roell Z100 (Zwick Roell, Ulm, Germany) test bench as described in ISO 7198:1998 [39]. Four 1 × 5 cm rectangular sheets for each matrix were cut and placed between holders at a distance of 2–2.5 cm. Tensile strength testing was performed at a rate of 10 mm·min^−1^ at room temperature (21–23 °C).

#### 2.3.2. Matrix Surface Characterization

The morphology of matrices were studied by scanning electron microscopy (SEM), as described earlier [35]. The size of the pores and the diameter of fibers were estimated from the SEM images according to ISO 7198:1998 [39]. To estimate the stability of the fiber structure, the matrices were incubated in PBS (Sigma-Aldrich, USA) or blood plasma (BP) at 37 °C for 27 days. After incubation, the matrices were rinsed with water, air-dried, and studied by SEM. To evaluate the structure after elongation, the matrices were fixed between clumps of the small device that expands the matrix and fixes it in an SEM camera. Matrices were fixed at conductive colloidal graphite (G303, Agar Scientific Ltd., Cambridge, UK) before gold sputter-coating.

#### 2.3.3. Additional Characterization of the Matrices

The contact angle was measured on a Drop Shape Analyzer–DS A25 (Kruss GmbH, Hamburg, Germany) using water as a solvent (drop volume, 1 µL; shooting speed, 160 frames per second).

The porosity of the matrices was evaluated according to the following formula:

Porosity (%) = [1 − Da/Dp] × 100

where Da = matrix weight/matrix volume and Dp is the PCL density.

### 2.4. Sirolimus Release Kinetics

To evaluate SRL release, 10 mm diameter disks (~0.785 cm^2^) were excised from the matrices by die cutting and placed in wells of a 48-well plate. Then, 400 µL of PBS or EDTA-stabilized BP was added to each well. The investigation was approved by the Local Ethical Committee of Center of Personalized Medicine, Institute of Chemical Biology and Fundamental Medicine of the Siberian Branch of the Russian Academy of Sciences (№1, 15.01.2016). The plate was sealed with a film (Microseal^®^ ‘B’ PCR Plate Sealing Film, adhesive, Bio-Rad, Hercules, CA, USA) to prevent drying, followed by incubation on a Titramax 1000 shaker (Heidolph, Schwabach, Germany) at 37 °C; platform rotation speed of 200 rpm. Moreover, 0.01% sodium azide was used to prevent bacterial growth. Two series of SRL release kinetics were studied. The matrices were incubated in PBS or BP for 20 min, 60 min, 3 h, 9 h, 27 h, 3 days, 9 days, and 27 days without (Series 1) or with (Series 2) medium replacement. For Series 2, the supernatant was removed at each time point, fresh solution was added, and the matrix was incubated in a fresh solution until the next time point. After incubation, the matrix was washed with H_2_O and air-dried at room temperature for SEM analysis. The radioactivity of the supernatants was measured in duplicate, as described above (Section 2.1).

The influence of matrix elongation on SRL release was evaluated as follows. The matrix was fixed in the clamps and then slowly stretched by a screw to double the measured distance. After stretching, the linear sizes of the matrix were measured and the amount of SRL per disk was calculated. The 10 mm diameter discs were then excised from the stretched matrix, and SRL release was estimated as described above. All experiments were performed in duplicate.

### 2.5. Statistical Analysis

The results were processed using Microsoft Excel 2010 and the Statistica 7.0 package (StatSoft Inc., Tulsa, OK, USA).

## 3. Results and Discussion

### 3.1. Preparation and Characterization of the SRL Matrices

^3^H-SRL was obtained by thermoactivated tritium exchange. ^3^H-SRL has the same Rf after RP-HPLC and TLC, suggesting a similar chemical structure of the compound. Purified ^3^H-SRL preparation with a specific radioactivity of 0.2 mCi/mL (~0.055 Ci/mmol) was thus obtained. The compound was homogenous according to the TLC data, being detected as a single spot on the autoradiograph with the expected Rf corresponding to unlabeled SRL. ^3^H-SRL was combined with unlabeled SRL to reach a dose of ~1.05 μg/cm^2^ SRL and a radioactivity of ~27,000 cpm/cm^2^.

ES is a very promising method based on the generation of polymer fibers from a blend of polymers and drugs in a strong electric field. The advantages of the ES method are the ability to obtain fibers with different diameters (from nm to µm) and ultrastructures, broad material suitability, and high cost effectiveness [30,31]. Several parameters have an influence on the structure and morphology of polymer fibers, including voltage, collector to needle distance, solution flow rate, polymer concentration, solvent type, dielectric constant, temperature, pressure, and moisture [31]. The solvent type, polymer concentration, and voltage strongly affect the resulting fiber diameter. Drug release and dissolution of the bioactive substance can be controlled by the structure of the polymer fibers. In this study, we used the same ES conditions to obtain all matrices.

Three different matrices were prepared using ES on a drum collector from solutions containing PCL, HSA, DMSO, SRL, and ^3^H-SRL. Electrospinning conditions were the same for all matrices: voltage—26.5 kV, feed rate—1 mL/h; nozzle to collector distance—20 cm.

Polymers used for drug-eluting stent coatings need to have mechanical characteristics and biological properties similar to those of natural vessels. The PCL scaffolds exhibit good mechanical properties, flexibility, and nontoxicity [40]. The average degradation time for PCL is about 2–3 years [33]. Earlier, we studied the structure of fibers produced by ES from PCL–HSA solutions [35]; like other authors, we demonstrated that HSA increases the hemocompatibility of blood-exposed surfaces [35,36,37]. The mechanical properties of PCL/HSA-containing matrices improved compared with pristine PCL, and HSA was tightly bound, with the fiber surface exposed for a long period of time because it is only partially accessible for protease hydrolysis [35]. HSA contains carboxylic and amine groups that promote cell attachment and spreading via integrin-binding sites, and can thus provide endothelization [40]. As such, HSA is the most abundant protein in human blood responsible for fatty acid transport and is the major binding protein for neutral and acidic drugs [41,42]. Its non-immunogenicity, high biocompatibility, and good biodegradability (as well as its binding of drugs) make HSA a prospective compound for DES coatings.

The physical properties of the obtained matrices are shown in Table 1. The tensile strength of the prepared matrices depend on the composition of the ES solution, demonstrating a typical two-phase curve with an extended plastic deformation region, starting from 7–10% to 250–300% and varying from 10.4 to 15 MPa (Table 1). These properties provide elongation without breakage during stent expansion, as well as a low residual load after the stent installation, as previously discussed [29].

**Figure 1:**
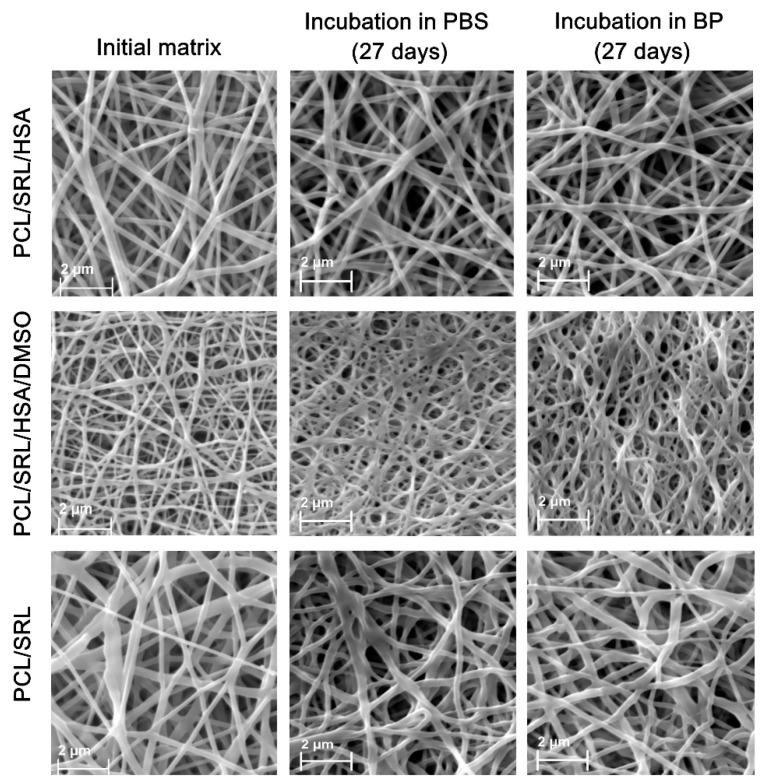
Scanning electron microscopy (SEM) images of the matrices incubated under different conditions. BP, blood plasma; PCL, polycaprolactone; SRL, sirolimus; HSA, human serum albumin: DMSO, dimethyl sulfoxide.

The structure of the matrices was studied by SEM. All matrices were composed of randomly oriented fibers. The composition of the ES solution influenced the fiber diameter and its variability (Figure 1). The matrices obtained from the blend containing 5% PCL, SRL, and 0.1 mM TEA were more heterogeneous in fiber diameter. The average diameter of the fibers made from the PCL/SRL/HSA and PCL/SRL solution was 0.27 and 0.36 µm, respectively (Table 1, Figure 1). The fiber diameter significantly decreased when DMSO was added (0.14 µm for the PCL/SRL/HSA/DMSO matrix). The SEM at a higher resolution reveals the smooth surface of the PCL fibers. The decrease in the fiber diameter for matrices containing DMSO was previously shown for the PCL-based matrices containing paclitaxel [29].

According to SEM data, incubation in PBS for 27 days did not alter the structure of the PCL/SRL/HSA and PCL/SRL matrices (Figure 1), while fiber fusion and thickening were observed when the PCL/SRL/HSA/DMSO matrix was incubated in PBS or BP for 27 days. A negligible thickening effect was also observed for the PCL/SRL matrix, where the thickness of the PCL/SRL/HSA/DMSO fibers increased almost twofold. There was no significant change in the structure of the PCL/SRL/HSA matrix during incubation. No significant weight change after 27 days of incubation was observed for all matrix types (data not shown). This means that, during the assayed time, there was no substantial matrix degradation. The incorporation of hydrophilic biodegradable components like HSA into the PCL matrices can potentially increase their rate of biodegradation, but we did not observe such an effect for 27 days [40].

The porosity of the matrices was estimated from the matrix weight and polymer density in the range of 60–65% (Table 1). No significant differences were observed for the matrices prepared from different blends. The presence of DMSO in the electrospinning solution resulted in a decrease not only in fiber diameter, but also in pore size. The PCL/SRL/HSA/DMSO matrix containing 3% DMSO had a pore diameter approximately 1.7-fold less than those without DMSO (Table 1, Figure 1). These data correlate with those previously obtained for PCL-based matrices containing paclitaxel [29].

The wettability of the matrices affected the cell distribution in the matrix and cell proliferation [40]. The polar amine and carboxyl groups in HSA might have improved the surface wettability of the matrices. HSA has a high water-binding capacity and can increase the hydrophilicity of materials [40]. Measurement of the contact angle is a commonly used technique to determine wettability. The water contact angles of the matrices are indicated in Table 1. The HSA-containing matrices (PCL/SRL/HSA and PCL/SRL/HSA/DMSO) show a lower contact angle compared with that of PCL/SRL. According to the literature data, hydrophilic matrices are more suitable for implants owing to their lower ability to adsorb proteins and induce inflammation [43,44].

### 3.2. Study of SRL Release

**Figure 2:**
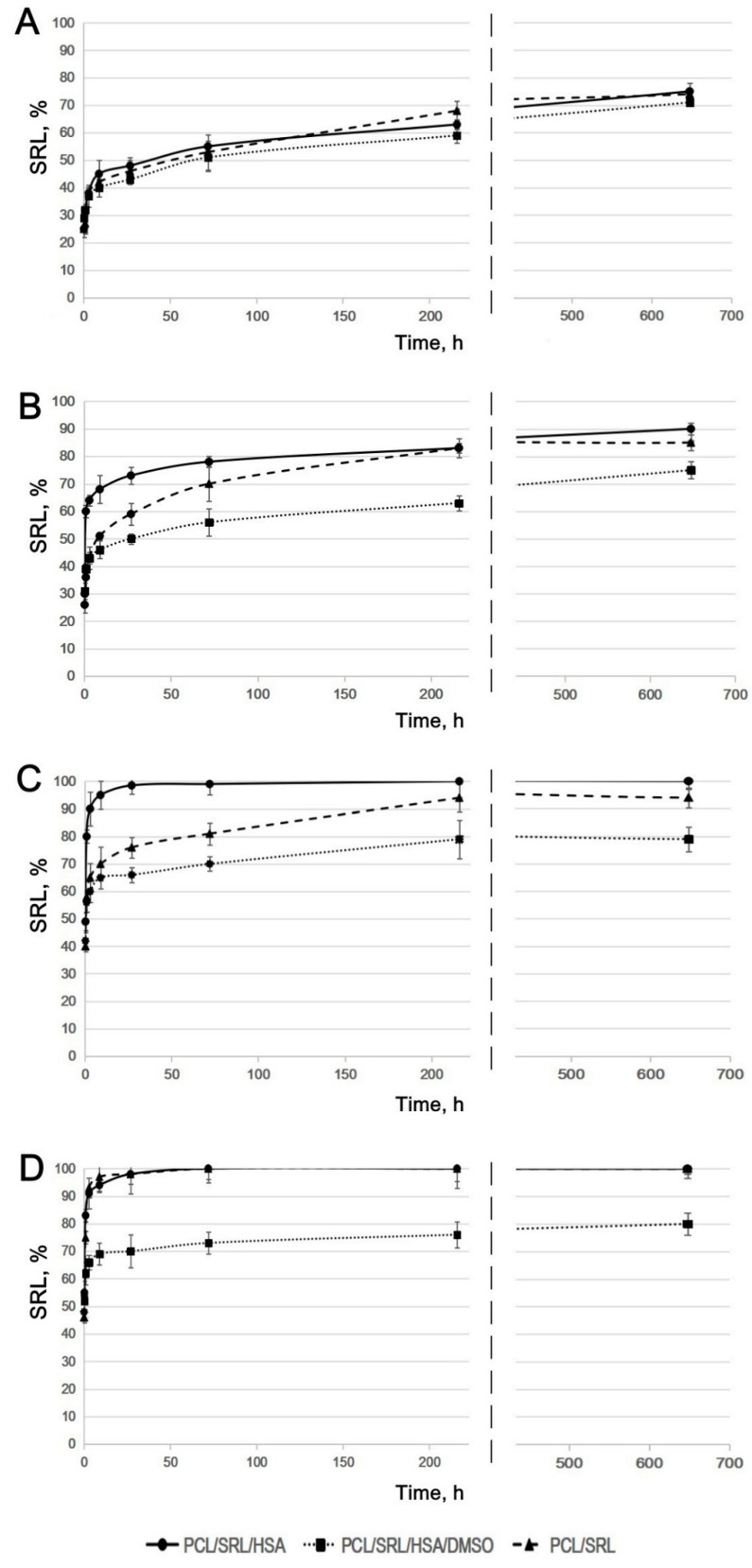
The kinetic curves of SRL release from the matrices. The matrices were incubated (**A**) in PBS without any medium replacement; (**B**) in PBS with medium replacement; (**C**) in BP without any medium replacement; and (**D**) in BP with medium replacement. The data are presented as the means of four independent measurements; the error of the mean did not exceed 7%.

The SRL release from PCL-based matrices was studied with (2) or without (1) a medium replacement to mimic SRL release in a biological system. The kinetic curves shown for PBS without any medium replacement were similar for all matrices (Supplementary materials, Table S1), although SRL release from the PCL/SRL/HSA/DMSO matrix was slightly slower, with the maximal release of 70% observed at 27 days (Figure 2A). The maximal release observed for the PCL/SRL/HSA and PCL/SRL matrices was 76% and 74%, respectively. When the matrices were incubated with replacement of PBS throughout, the rate of SRL release from the PCL/SRL/HSA/DMSO matrix was significantly slower, with a maximal level of 75% for 27 days (Figure 2B). Meanwhile, the PCL/SRL/HSA and PCL/SRL matrices released 75% SRL during the first three days, and reached maximal levels of 90% and 85%, respectively.

It was shown earlier that paclitaxel is distributed both throughout the volume and on the surface of the fibers in PCL-based matrices [29]. In this work, the SRL distribution seems to be very similar to that of paclitaxel, because we used the same method to obtain matrices. Desorption of SRL from the matrix surface limits the rate of establishing the equilibrium between SRL on the surface and SRL in the solution. This process is the result of the low solubility of SRL in water (2.6 mg/L) [45]. Removing the medium with dissolved SRL led to the additional desorption of SRL from the matrix surface into the solution, thus the amount of dissolved SRL increased. The presence of ions and SRL-binding molecules can also increase the solubility of SRL in PBS and BP.

When incubated in BP, SRL was completely released from the PCL/SRL/HSA matrix after 27 h (Figure 2C). The kinetic curve for the PCL/SRL matrix showed a slower SRL release, with saturation achieved over 6–9 days, and a maximal level of 96% was observed at 27 days.

HSA acts as the main transporter of SRL in blood and tissues. HSA has two ligand-specific binding sites for drugs, site I and site II (high-affinity binding and low-affinity binding, respectively) [42,46,47]. SRL binds to albumin at site I more often than at site II. At low BP concentrations, SRL binds to site I. At higher PB concentrations, both sites participate in SRL binding. The affinity association constant for SRL binding to HSA was determined to be 3.99 × 10^5^ M^−1^ [42,46]. Apparently, the SRL resorption on the matrix surface is hindered by the binding of SRL with HSA in BP. It is also possible that other biomolecules in plasma interact with SRL.

Incubation with a BP replacement resulted in an increase in SRL release from the PCL/SRL matrix of up to 100%. According to the SEM data, there were no changes in the fiber structure of PCL/SRL matrices during the 27-day incubation in PBS or BP (Figure 1). This means that long-term SRL release is not associated with matrix structure reorganization or fiber degradation. According to the kinetic study, the PCL/SRL/HSA and PCL/SRL matrices are not capable of prolonged SRL delivery because of rapid SRL release (100% in three days) in BP. The changes in fiber structure and/or SRL distribution inside the fibers are necessary to obtain matrices suitable for prolonged SRL release. Addition of DMSO to the electrospinning blend enabled to obtain porous fibers and retain SRL in the fibers. DMSO is nontoxic, has a boiling point of 189 °C, and dissolves SRL. Moreover, as shown previously, the addition of 3% DMSO to the ES blend significantly reduced paclitaxel release in both PBS and BP [29]. As indicated above, the addition of 3% DMSO to the ES blend resulted in both decreased fiber diameter and pore size. In these matrices, the fiber diameters nearly doubled over the 27 days of incubation owing to the water absorption by HSA. The change in structure of the freshly obtained fibers and those of the fibers after incubation of the PCL/SRL/HSA/DMSO matrices, as well as for the initial and long-term incubation, induced redistribution of SRL in the fibers and obviously affected SRL release. The lowest rate of SRL release in BP (as well as that in PBS) was observed for the matrix containing DMSO. The matrix produced from a 5% PCL/SRL/10% HSA/3% DMSO blend released a maximum of 80% SRL over 27 days, both with and without medium replacement. The data obtained demonstrate the importance of the PCL/SRL/HSA/DMSO matrix as a prospective material for DES coating.

### 3.3. Influence of Matrix Elongation on SRL Release

**Figure 3:**
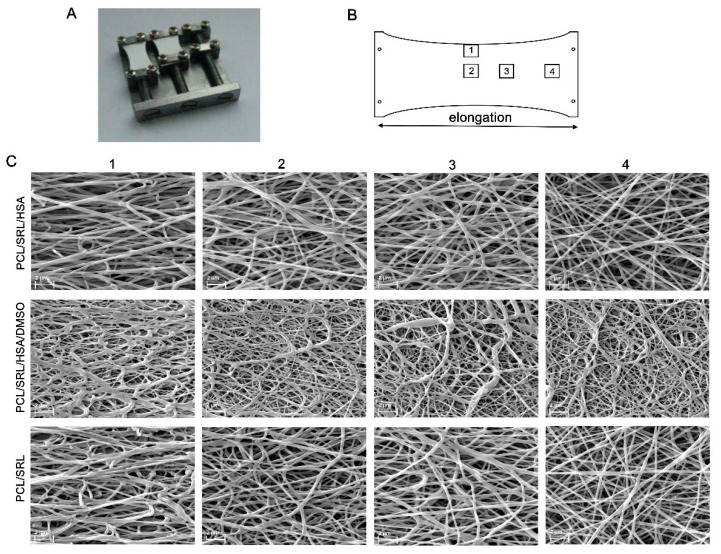
SEM images of the expanded matrices. (**A**) A device allowing one to expand the matrices and fix them in a sputter coater and SEM camera. (**B**) Location of the areas studied by SEM. (**C**) SEM images of different areas of the matrices.

**Figure 4:**
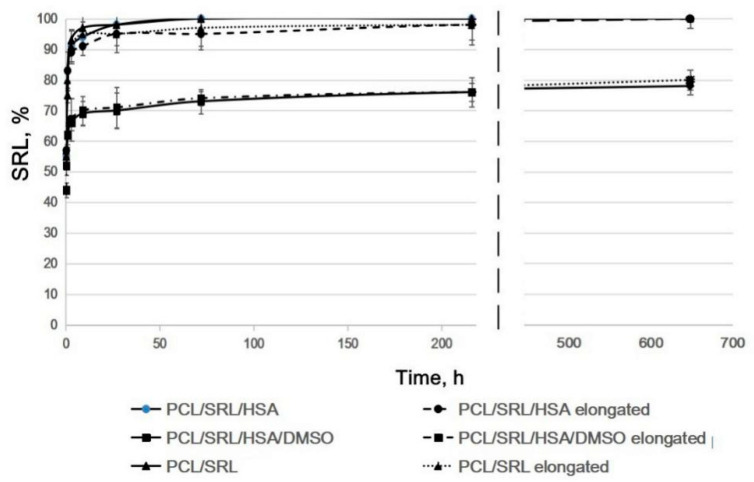
The kinetic curves of SRL release from the expanded matrices. Incubation of the matrices in BP with medium replacement. The data are presented as the means of four independent measurements; error of the mean did not exceed 7%.

Considering the twofold elongation of the matrices covering the vascular stents during their installation, the effect of the deformation of the matrices on drug release was studied. To study the influence of twofold elongation on the matrix structures, we used a device that expands the matrix and fixes it in an SEM camera (Figure 3A). This type of deformation differs from the deformation during stent implantation, whereupon uniform deformation of the tube walls occurs, but can simulate the influence of deformation on fiber location and structure. Different areas of the matrices were studied by SEM owing to unequal deformation of the matrices (the maximal deformation was in the center and the minimal deformation was near the clamps) (Figure 3B). In the central area (Figure 3B, 1 and 2; Figure 3C columns 1 and 2), SEM demonstrated that twofold elongation did not lead to a disruption of the fibers, although it was accompanied by the orientation of the fibers along the force vector and compaction of the fiber stack. The structure of the matrix near the clamps with minimal stretching (Figure 3B, 4; Figure 3C column 4) resembles an intact matrix (Figure 1). The intermediate area (Figure 3B, 3; Figure 3C column 3) differs in that fibers are only slightly oriented. After twofold elongation of the matrices and removal of the load, the linear dimensions were reduced by 10–17% (compared with twofold elongation), depending on the type of matrix. According to the SEM data, all obtained matrices are able to withstand elongation during stent installation.

As shown in Figure 4, SRL release from the parent and the twofold expanded matrices were very similar for all matrices under study (Supplementary materials, Table S1). The PCL/SRL/HSA and PCL/SRL matrices present very similar curves of SRL release. SRL was completely released from the PCL/SRL/HSA and PCL/SRL/TEA matrices, with maximal release in the first 27 h, whereas SRL release from the PCL/SRL/HSA/DMSO matrix was significantly slower, with a maximal level of 80% for 27 days. Data on the SRL release from the matrices with BP replacement demonstrate that twofold elongation barely changes the release kinetics of the matrices (when normalized to the linear/weight/volume characteristics of the matrices). Nevertheless, it is necessary to account for the changes in the areas of the matrices after stent installation when calculating the dose of the drug introduced into the matrix, considering the approximately 1.8-fold increase in the linear size, and thus the decrease in the specific content of the drug in the covered square.

## 4. Conclusions

The structure and physicochemical characteristics of the ES matrices obtained from the solutions of PCL with SRL in HFIP and their blends with HSA, DMSO, and TEA were studied. The matrix produced from 5% PCL/SRL/10% HSA/3% DMSO was shown to be the most suitable for bare-metal stent coating because it is sufficiently strong, exhibited long-term SRL release kinetics, and is thus expected to maintain a concentrations of SRL in the vascular wall that are suitable for antiproliferative activity over a long period. The use of BP increased SRL release. We showed that all matrices can release SRL without fiber degradation. The twofold elongation of the matrices barely changed the SRL release kinetics. Therefore, PCL-based matrices containing HSA, SRL, and DMSO are a promising material for vascular stent coating with prolonged delivery of SRL.

## Figures and Tables

**Table 1:** Physical properties of the matrices. PCL, polycaprolactone; SRL, sirolimus; HSA, human serum albumin: DMSO, dimethyl sulfoxide.

Matrix Type	Thickness (µm)	Strength (MPa)	Mass Per Unit Area (mg/cm^2^)	Fiber Diameter (µm)	Pore Diameter (µm)	Porosity (%)	Contact Angle (°)
5% PCL/SRL/10% HSA	111	10.45 ± 1.48	2.51	0.27 ± 0.8	1.94 ± 0.33	63.3	117.63 ± 2.72
5% PCL/SRL/10% HSA/3%DMSO	125	12.41 ± 3.11	2.47	0.14 ± 0.05	1.12 ± 0.18	64.7	109.52 ± 2.22
5% PCL/SRL	114	15.04 ± 2.50	2.44	0.36 ± 0.09	2.11 ± 0.34	60.9	127.37 ± 3.41

## Supplementary Materials

The following are available online at https://www.mdpi.com/1996-1944/13/12/2692/s1, Table S1: Release curves comparison using Fit factors f1 and f2; Table S2: Release curves comparison using Fit factors f1 and f2. Comparison of kinetic curves of SRL release from the parent and the two-fold expanded matrices. Incubation of the matrices in BP with medium replacement.
