# Supplementary material for: Sirolimus-Eluting Electrospun-Produced Matrices as Coatings for Vascular Stents: Dependence of Drug Release on Matrix Structure and Composition of the External Environment
Source: Materials (Basel). 2020 Jun 12;13(12):2692. doi: 10.3390/ma13122692 (PMC7345069; doi:10.3390/ma13122692)
Supplement: Supplementary file 1 [file materials-13-02692-s001.pdf]

Table S1. Release curves comparison using Fit factors f1 and f2.

**Incubation of the matrices with PBS without any medium replacement**			
**Matrix type**	**5% PCL/SRL/10% HSA**	**5% PCL/SRL/10% HSA/3%DMSO**	5% PCL/SRL
5% PCL/SRL/10% HSA	-	f1 = 7.3<br>f2 = 70.2	f1 = 4.5<br>f2 = 76.8
5% PCL/SRL/10% HSA/3%DMSO	f1 = 7.3<br>f2 = 70.2	-	f1 = 8.0<br>f2 = 67.7
5% PCL/SRL	f1 = 4.5<br>f2 = 76.8	f1 = 8.0<br>f2 = 67.7	-
Incubation of matrices with PBS with medium replacement			
Matrix type	5% PCL/SRL/10% HSA	5% PCL/SRL/10% HSA/3%DMSO	5% PCL/SRL
5% PCL/SRL/10% HSA	-	f1 = 26.6<br>f2 = 35.6	f1 = 17.0<br>f2 = 42.4
5% PCL/SRL/10% HSA/3%DMSO	f1 = 26.6<br>f2 = 35.6	-	f1 = 16.4<br>f2 = 49.4
5% PCL/SRL	f1 = 17.0<br>f2 = 42.4	f1 = 16.4<br>f2 = 49.4	-
Incubation of the matrices with BP without any medium replacement			
Matrix type	5% PCL/SRL/10% HSA	5% PCL/SRL/10% HSA/3%DMSO	5% PCL/SRL
5% PCL/SRL/10% HSA	-	f1 = 27.3<br>f2 = 29.7	f1 = 18.8<br>f2 = 36.7
5% PCL/SRL/10% HSA/3%DMSO	f1 = 27.3<br>f2 = 29.7	-	f1 = 12.6<br>f2 = 50.9
5% PCL/SRL	f1 = 18.8<br>f2 = 36.7	f1 = 12.6<br>f2 = 50.9	-
Incubation of the matrices with BP with medium replacement			
Matrix type	5% PCL/SRL/10% HSA	5% PCL/SRL/10% HSA/3%DMSO	5% PCL/SRL
5% PCL/SRL/10% HSA	-	f1 = 24.9<br>f2 = 32.0	f1 = 1.8<br>f2 = 74.3

5% PCL/SRL/10% HSA/3%DMSO	f1 = 24.9<br>f2 = 32.0	-	f1 = 31.0<br>f2 = 32.0
5% PCL/SRL	f1 = 1.8<br>f2 = 74.3	f1 = 31.0<br>f2 = 32.0	-

Table S2. Release curves comparison using Fit factors f1 and f2. Comparison of kinetic curves of SRL release from the parent and the two-fold expanded matrices. Incubation of the matrices in BP with medium replacement.

**Matrix type**	**5% PCL/SRL/10% HSA expanded**	**5% PCL/SRL/10% HSA/3%DMSO expanded**	**5% PCL/SRL expanded**
5% PCL/SRL/10% HSA	f1 = 2.4<br>f2 = 77.6	-	-
5% PCL/SRL/10% HSA/3%DMSO	-	f1 = 2.6<br>f2 = 75.0	-
5% PCL/SRL	-	-	f1 = 2.6<br>f2 = 76.9

Fit factors were calculated as:

$$f_1 = \{[\Sigma_{t=1}{}^{n} | R_t - T_t |]/[\Sigma_{t=1}{}^{n} R_t]\} \cdot 100$$
$$f_2 = 50 \cdot \log \{[1+(1/n)\Sigma_{t=1}{}^{n} (R_t - T_t)^2]^{-0.5} \cdot 100\}$$

Rt and Tt are the cumulative percentage released at each of the selected n time points of the reference and test product respectively. The difference factor f1 is proportional to the average difference between the two profiles, where as similarity factor f2 is inversely proportional to the average squared difference between the two profiles, with emphasis on the larger difference among all the time-points. The f1 value is 0 when the test and the reference profiles are identical and increases proportionally with the dissimilarity between the two profiles. The f2 value is between 0 and 100. f2 value between 50-100 indicates similarity between two dissolution profiles.

References

Shah, V.P.; Tsong, Y.; Sathe, P.; Williams, R.L. Dissolution Profile Comparison Using Similarity Factor, f2. *Dissolution Technologies*, **1999**, *6*, 15.
